# Fructose Intake Impairs the Synergistic Vasomotor Manifestation of Nitric Oxide and Hydrogen Sulfide in Rat Aorta

**DOI:** 10.3390/ijms22094749

**Published:** 2021-04-30

**Authors:** Andrea Berenyiova, Samuel Golas, Magdalena Drobna, Martina Cebova, Sona Cacanyiova

**Affiliations:** Institute of Normal and Pathological Physiology, Centre of Experimental Medicine Slovak Academy of Sciences, 841 04 Bratislava, Slovakia; samuel.golas@savba.sk (S.G.); magdalena.drobna@savba.sk (M.D.); martina.cebova@savba.sk (M.C.); sona.cacanyiova@savba.sk (S.C.)

**Keywords:** fructose, Wistar Kyoto rats, thoracic aorta, nitric oxide, hydrogen sulfide

## Abstract

In this study, we evaluated the effect of eight weeks of administration of 10% fructose solution to adult Wistar Kyoto (WKY) rats on systolic blood pressure (SBP), plasma and biometric parameters, vasoactive properties of the thoracic aorta (TA), NO synthase (NOS) activity, and the expression of enzymes producing NO and H_2_S. Eight weeks of fructose administration did not affect SBP, glycaemia, or the plasma levels of total cholesterol or low-density and high-density lipoprotein; however, it significantly increased the plasma levels of γ-glutamyl transferase and alanine transaminase. Chronic fructose intake deteriorated endothelium-dependent vasorelaxation (EDVR) and increased the sensitivity of adrenergic receptors to noradrenaline. Acute NOS inhibition evoked a reduction in EDVR that was similar between groups; however, it increased adrenergic contraction more in fructose-fed rats. CSE inhibition decreased EDVR in WKY but not in fructose-fed rats. The application of a H_2_S scavenger evoked a reduction in the EDVR in WKY rats and normalized the sensitivity of adrenergic receptors in rats treated with fructose. Fructose intake did not change NOS activity but reduced the expression of eNOS and CBS in the TA and CSE and CBS in the left ventricle. Based on our results, we could assume that the impaired vascular function induced by increased fructose intake was probably not directly associated with a decreased production of NO, but rather with impairment of the NO–H_2_S interaction and its manifestation in vasoactive responses.

## 1. Introduction

Currently, significant changes in nutritional habits present an important factor encroaching into the equilibrium of different signaling pathways in the body. Excessive intake of added sugars, mainly as sugar-sweetened beverages, has been implicated in the development of obesity and metabolic disturbances, increasing the risk of cardiovascular disease mortality. Two major sweeteners are predominantly used in the food and beverage industry: sucrose, a disaccharide containing 50% fructose and 50% glucose; and high-fructose corn syrup, which mainly consists of 55% fructose and 45% glucose [1,2].

Fructose is a natural, simple sugar found in fruits and honey that is responsible for their sweet taste. Due to its specific metabolism, predominantly in the liver, fructose is more lipogenic than glucose. Long-term application of this saccharide at different concentrations in food or drinking water has been used to induce metabolic disorders in experimental models [3]. Animals chronically receiving high amounts of fructose developed hypertriglyceridemia, insulin resistance, hyperinsulinemia, and often obesity and hypertension [4,5,6]. Zemancikova and Torok (2014) [7] also reported that eight weeks of fructose administration to adult Wistar rats induced metabolic changes associated with an increase in plasma glucose and lipids, enlargement of the liver, significant blood pressure elevation, and reduction in endothelium-derived vasorelaxation. The authors also pointed out the specific role of NO/NOS signaling in these processes, because simultaneous treatment with a NOS inhibitor and fructose did not evoke the abovementioned pathological changes.

Literary data show that another gaseous molecule, endogenously synthesized hydrogen sulfide (H_2_S), is involved in the same biological processes as NO, including the vasomotor responses of the arterial wall, neurotransmission, control of infectious processes, and insulin release [8,9]. Pan et al. (2014) [10] demonstrated that a high-saccharide environment inhibits the expression of one of the H_2_S-producing enzymes, cystathionine-γ-lyase (CSE), in adipocytes. Several studies have also suggested that high glucose intake significantly increases the secretion of pro-inflammatory cytokines, including tumor necrosis factor-α (TNF-α), IL-6, and monocyte chemoattractant protein-1 [11,12], and the involved mechanism is partly related to inhibition of the CSE/H_2_S system.

Based on this, we can assume that the endogenous NO and H_2_S signaling pathways could be involved in the metabolic disorders caused by high saccharide intake. However, the data related to their vasoregulatory mechanisms in this circumstance are still limited. The main goal of the present study was to describe the effect of eight weeks of fructose administration to adult Wistar Kyoto (WKY) rats on the vasoactive properties of the thoracic aorta, with special emphasis on the participation of NO and H_2_S signaling pathways. Such observations have so far not been made. We hypothesized that the increased intake of fructose induces metabolic syndrome-like disorders that could probably modulate the NO and H_2_S signaling pathways and their interaction.

## 2. Results

### 2.1. Blood Pressure, Cardiac Hypertrophy, Weight Gain, and Fluid and Food Intake

**Figure 1:**
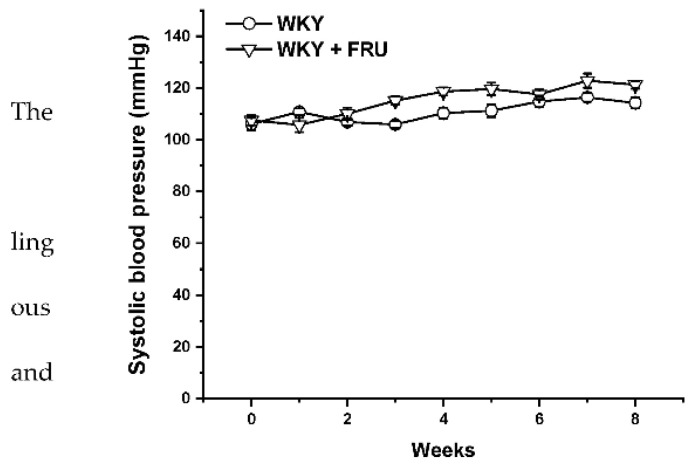
Time course of systolic blood pressure in Wistar Kyoto (WKY) and in Wistar Kyoto rates treated with 10% fructose solution (WKY + FRU) (*n* = 6). Data are expressed as the mean ± SEM.

**Figure 2:**
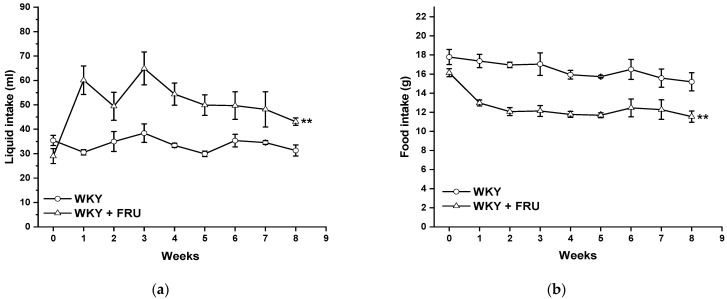
Time course of liquid (**a**) and food (**b**) intake in Wistar Kyoto (WKY) and in Wistar Kyoto rats treated with 10% fructose solution (WKY + FRU) (*n* = 6). Data are expressed as the mean ± SEM. ** *p* < 0.01 vs. WKY.

Long-term increased fructose intake had a significant effect on the values of systolic blood pressure (SBP) (F_(1;219)_ = 14.25; *p* = 2.09 × 10^−4^); however, the statistical analysis did not reveal any differences between the experimental groups in individual weeks (Figure 1). Fructose treatment had no impact on the parameters of heart trophicity; neither the ratio of heart weight to body weight nor the tibial length to body weight were changed after fructose treatment (Table 1). Surprisingly, the eight-week fructose treatment did not affect the weight gain or adiposity (expressed as a ratio of the retroperitoneal adipose tissue weight to the tibia length) of the animals (Table 1). Although the animals receiving the fructose solution had an increased average weekly liquid intake, they had a significantly lower weekly food intake than the control group (Figure 2a,b).

### 2.2. Plasma Parameters

The levels of triacylglycerols (TAG), high-density cholesterol (HDL), alanine aminotransferase (ALT), gamma glutamyl transferase (γ-GT), cystatin C (CYS), creatinine (CRE) and glucose (GLU) were determined in plasma samples from truncal blood. Fructose treatment had no impact on the TAG, HDL, CYS, or GLU levels, but significantly increased the ALT and γ-GT levels, indicating liver injury (Table 2). Moreover, there was a non-significant trend of CRE level increase (*p* < 0.07), suggesting an initial renal impairment.

### 2.3. Vasoactive Responses

**Figure 3:**
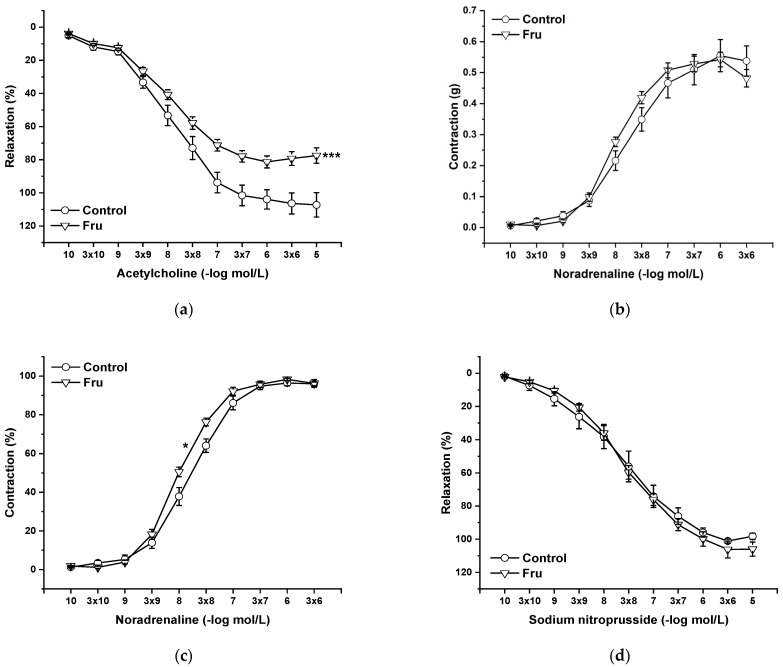
The effect of chronic increased fructose intake on the endothelium-derived vasorelaxation (**a**), adrenergic contraction (**b**), sensitivity of the adrenergic receptors (**c**), and vasorelaxant effect of sodium nitroprusside (**d**). Control—isolated thoracic aorta of Wistar Kyoto rats; Fru—isolated thoracic aorta of Wistar Kyoto rats treated with 10% fructose solution (*n* = 6). Data are expressed as the mean ± SEM. * *p* < 0.05; *** *p* < 0.001 vs. Control.

Endothelial functions were tested by the application of cumulative doses of acetylcholine (Ach, 10^−10^ to 10^−5^ mol/L), which induces vasorelaxation mediated by NO produced in endothelial cells. Chronic fructose intake significantly reduced endothelium-dependent vasorelaxation (Figure 3a). Cumulative application of exogenous noradrenaline (NA, 10^−10^ to 3 × 10^−6^ mol/L) induced vasoconstriction in a concentration-dependent manner by activating adrenergic receptors. Although the absolute force of contractile responses was not affected by the treatment (Figure 3b), it significantly increased the sensitivity of the adrenergic receptors (F_(1;258)_ = 11.53; *p* = 7.8 × 10^−4^) (Figure 3c). The vasorelaxant effect of the exogenous NO donor sodium nitroprusside (NS, 10^−10^ to 10^−5^ mol/L) was not affected by chronic fructose intake (Figure 3d).

**Figure 4:**
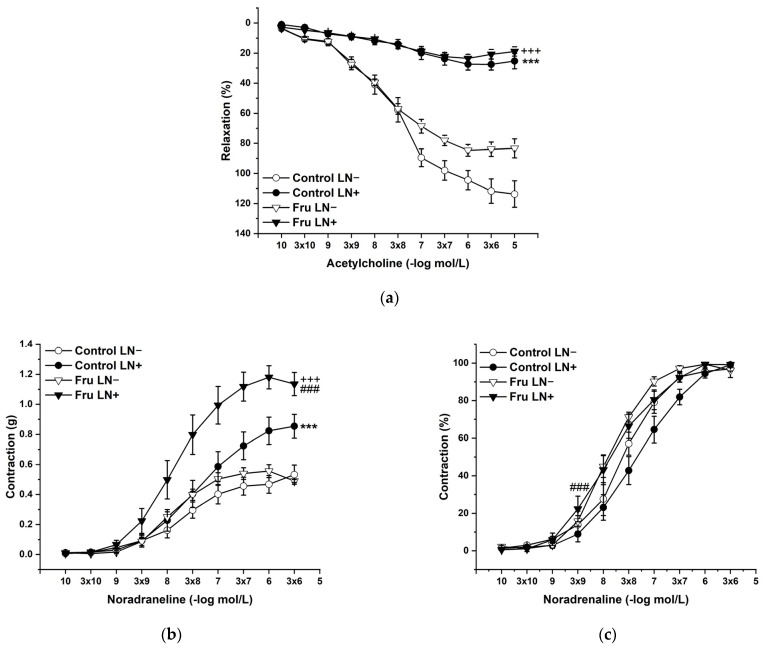
The participation of the endogenous NO in the endothelium-derived vasorelaxation (**a**), adrenergic contraction (**b**), and sensitivity of the adrenergic receptors (**c**). Control LN−: isolated thoracic aorta of Wistar Kyoto rats before N^G^-nitro-L-arginine methylester (LN 10^−4^ mol/L) incubation; Control LN+: isolated thoracic aorta of Wistar Kyoto rats after LN incubation; Fru LN−: isolated thoracic aorta of Wistar Kyoto rats treated with 10% fructose solution before LN incubation; Fru LN+: isolated thoracic aorta of Wistar Kyoto rats treated with 10% fructose solution before LN incubation (*n* = 6). Data are expressed as the mean ± SEM. *** *p* < 0.001 vs. Control LN-; +++ *p* < 0.001 vs. Fru LN-; ### *p* < 0.001 vs. Control LN+.

To evaluate the participation of endogenous NO in the basic vasoactive responses, we incubated the aortic rings with N^G^-nitro-L-arginine methylester (LN; 10^−4^ mol/L). LN incubation significantly inhibited endothelium-dependent vasorelaxation to the same degree in both experimental groups (Figure 4a). On the other hand, acute LN treatment increased the contractile response to exogenous NA, and this increase was significantly higher after fructose treatment than in control animals. Not only the treatment (F_(1;218)_ = 34.62; *p* = 2.08 × 10^−8^), but also the applied inhibitor (F_(1;218)_ = 90.62; *p* = 0) had a significant effect on these responses; moreover, a synergistic interaction between the treatment and the inhibitor (F_(1;218)_ = 14.71; *p* = 1.77 × 10^−4^) was proven (Figure 4b). The sensitivity of the adrenergic receptors was affected by the treatment (F _(1;218)_ = 18.46; *p* = 2.83 × 10^−5^) and the inhibitor (F _(1;218)_ = 4.15; *p* = 0.04), which were generally caused by the increased sensitivity in fructose-fed rats (Figure 4c).

**Figure 5:**
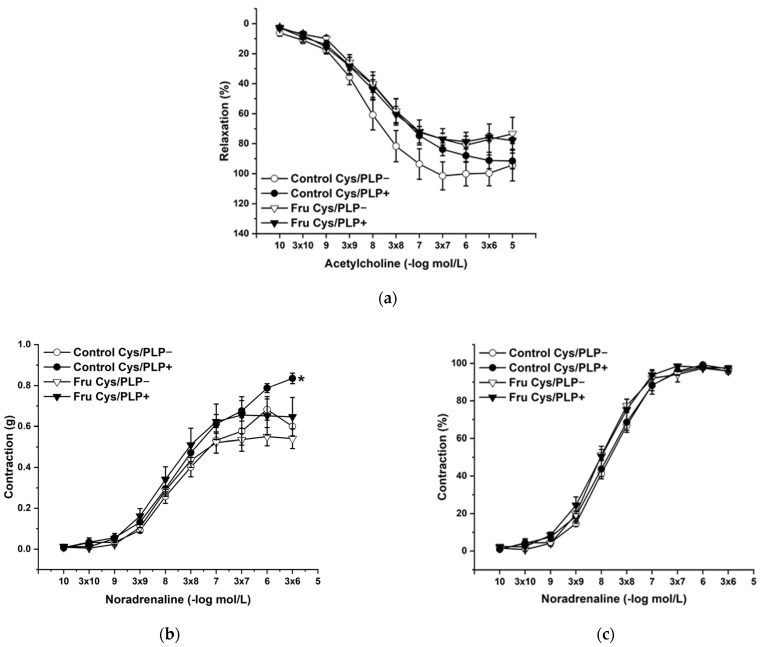
The effect of the incubation with a precursor and a cofactor of the endogenous H_2_S synthesis on the endothelium-derived vasorelaxation (**a**), adrenergic contraction (**b**) and sensitivity of the adrenergic receptors (**c**). Control Cys/PLP−: isolated thoracic aorta of Wistar Kyoto rats before L-cysteine (Cys; 5 × 10^−3^ mol/L) and pyridoxal 5′-phosphate (PLP; 2 × 10^−3^ mol/L) incubation; Control Cys/PLP+: isolated thoracic aorta of Wistar Kyoto rats after Cys/PLP incubation; Fru Cys/PLP−: isolated thoracic aorta of Wistar Kyoto rats treated with 10% fructose solution before Cys/PLP incubation; Fru Cys/PLP+: isolated thoracic aorta of Wistar Kyoto rats treated with 10% fructose solution before Cys/PLP incubation (*n* = 6). Data are expressed as the mean ± SEM. * *p* < 0.05 vs. Control Cys/PLP−.

**Figure 6:**
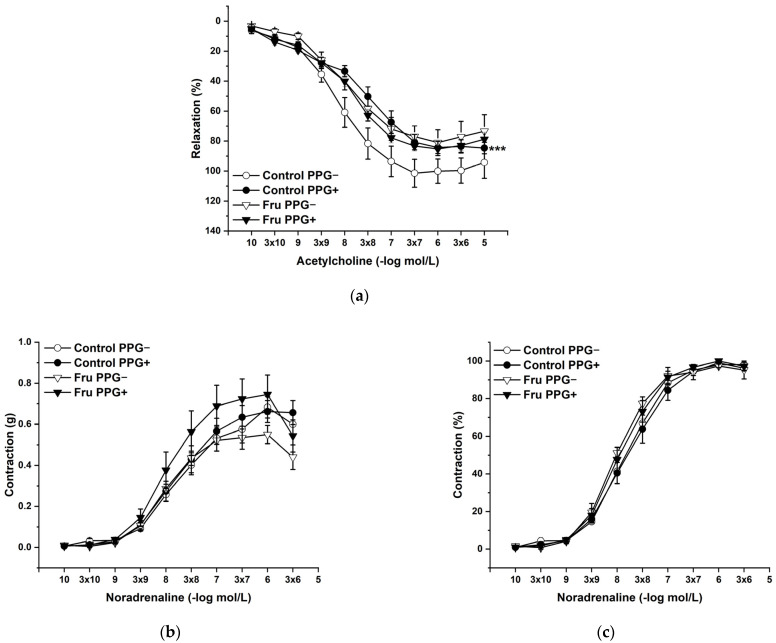
The participation of the cystathionine γ-lyase-derived H_2_S on the endothelium-derived vasorelaxation (**a**), adrenergic contraction (**b**) and sensitivity of the adrenergic receptors (**c**). Control PPG−: isolated thoracic aorta of Wistar Kyoto rats before DL-propargylglycine (PPG; 10^−2^ mol/L) incubation; Control PPG+: isolated thoracic aorta of Wistar Kyoto rats after PPG incubation; Fru PPG−: isolated thoracic aorta of Wistar Kyoto rats treated with 10% fructose solution before PPG incubation; Fru PPG+: isolated thoracic aorta of Wistar Kyoto rats treated with 10% fructose solution before PPG incubation (*n* = 6). Data are expressed as the mean ± SEM. *** *p* < 0.001 vs. Control PPG−.

**Figure 7:**
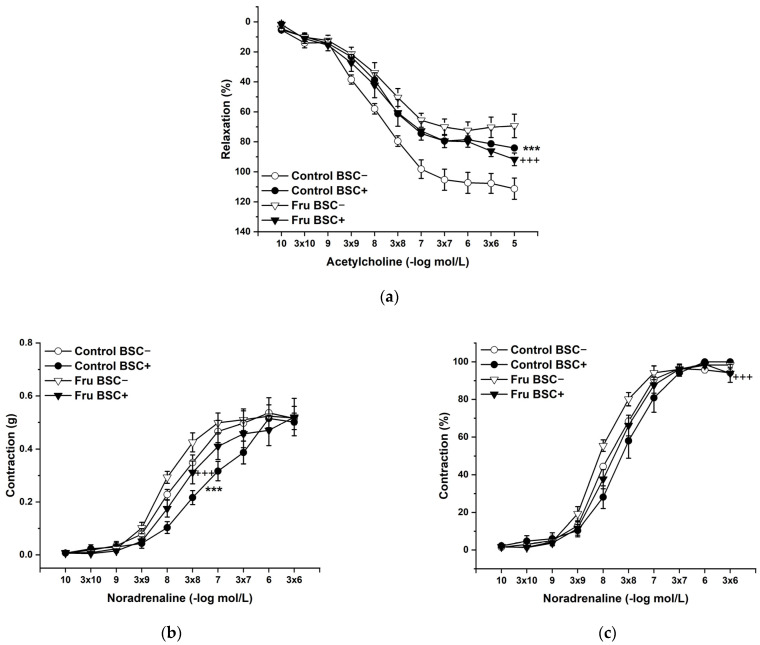
The effect of a H_2_S scavenger on the endothelium-derived vasorelaxation (**a**), adrenergic contraction (**b**), and sensitivity of the adrenergic receptors (**c**). Control BSC−: isolated thoracic aorta of Wistar Kyoto rats before bismuth (III) subsalicylate (BSC; 10^−6^ mol/L) incubation; Control BSC+: isolated thoracic aorta of Wistar Kyoto rats after BSC incubation; Fru BSC−: isolated thoracic aorta of Wistar Kyoto rats treated with 10% fructose solution before BSC incubation; Fru BSC+: isolated thoracic aorta of Wistar Kyoto rats treated with 10% fructose solution before BSC incubation (*n* = 6). Data are expressed as the mean ± SEM. *** *p* < 0.001 vs. Control BSC−; +++ *p* < 0.001 vs. Fru BSC−.

L-Cysteine (Cys; 5 × 10^−3^ mol/L), a precursor of endogenous H_2_S production, pyridoxal 5’-phosphate (PLP; 2 × 10^−3^ mol/L), a cofactor of cystathionine-γ-lyase (CSE), and cystathionine β-synthase (CBS) were added to the organ bath. Cys and PLP treatment did not induce any changes in endothelium-dependent vasorelaxation, but had a significant effect on the maximal force of the adrenergic contraction in the control group (F(_1;240_) = 10.54; *p* = 0.001; Figure 5a–c). Acute treatment with DL-propargylglycine (PPG; 10^−2^ mol/L) significantly decreased the vasorelaxant response to Ach in untreated rats and did not affect the contractile response to NA (Figure 6a–c), whereby the treatment–inhibitor interaction revealed no effect on these responses. The incubation with a H_2_S scavenger, namely, bismuth (III) subsalicylate (BSC; 10^−6^ mol/L), significantly reduced endothelium-dependent vasorelaxation in control rats and increased the vasorelaxation in rats receiving fructose solution (Figure 7a); moreover, the treatment–concentration–scavenger interaction had a significant effect on this response (F(_1;202)_ = 1.97; *p* = 0.032). The fructose treatment and the scavenger application also affected the adrenergic contractile (F_(1;228)_ = 6.62; *p* = 0.013; resp. F_(1;228)_ = 24.88; *p* = 1.37 × 10^−6^) responses and the sensitivity of the adrenergic receptors (F_(1;228)_ = 5.32; *p* = 0.022; resp. F_(1;228)_ = 12.54; *p* = 4.99 × 10^−4^) (Figure 7b,c). 

**Figure 8:**
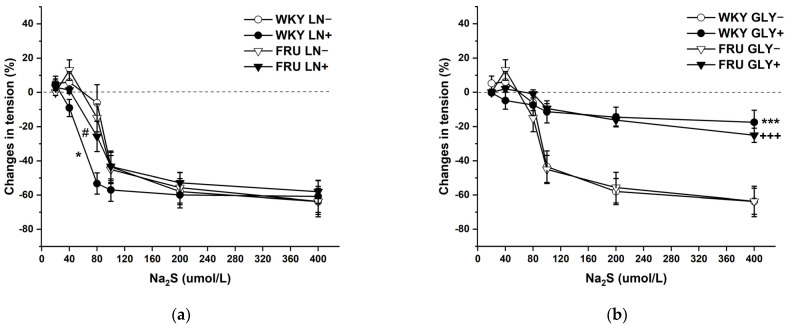
The dual vasoactive effect of H_2_S donor and the participation of the endogenous NO (**a**) and K_ATP_ channels (**b**) in the vasoactive responses. Control LN−: isolated thoracic aorta of Wistar Kyoto rats before N^G^-nitro-L-arginine methylester (LN 10^−4^ mol/L) incubation; Control LN+: isolated thoracic aorta of Wistar Kyoto rats after LN incubation; Fru LN−: isolated thoracic aorta of Wistar Kyoto rats treated with 10% fructose solution before LN incubation; Fru LN+: isolated thoracic aorta of Wistar Kyoto rats treated with 10% fructose solution before LN incubation; Control GLY−: isolated thoracic aorta of Wistar Kyoto rats before glibenclamide (GLY, 10^−4^ mol/L) incubation; Control GLY+: isolated thoracic aorta of Wistar Kyoto rats after GLY incubation; Fru GLY−: isolated thoracic aorta of Wistar Kyoto rats treated with 10% fructose solution before GLY incubation; Fru GLY+: isolated thoracic aorta of Wistar Kyoto rats treated with 10% fructose solution before GLY incubation (*n* = 6). Data are expressed as the mean ± SEM. * *p* < 0.05; *** *p* < 0.001 vs. Control LN− resp. Control GLY−; +++ *p* < 0.001 vs. Fru GLY−; # *p* < 0.05 vs. Control LN+.

The cumulative application of the H_2_S donor (Na_2_S·9H_2_O; 20, 40, 80, 10, 200, and 400 μmol/L) on NA-pre-contracted TA rings induced a dual effect in the experimental groups: lower concentrations of Na_2_S induced contraction, whereas higher concentrations evoked vasorelaxation of the arterial wall (Figure 8a,b). Chronic increased fructose intake had no effect on the vasoactive responses induced by the H_2_S donor. To evaluate the participation of endogenous NO in these responses, aortic segments were first incubated with LN (10^−4^ mol/L). Acute NO deficiency significantly increased the relaxant part of the dual vasoactive response to the H_2_S donor in control WKY rats, but not in rats treated with fructose solution (Figure 8a). The K_ATP_ channel inhibitor glibenclamide (GLY, 10^−4^ mol/L) significantly reduced the maximum of H_2_S donor-induced vasorelaxation similarly in both experimental groups, demonstrating a crucial role of hyperpolarization in these vasoactive responses (Figure 8b), which was unaffected by fructose treatment. Both LN and GLY incubation had a significant (F_(1;121)_ = 5.47; *p* = 0.02; F_(1;122)_ = 59.87; *p* = 8.38 × 10^−12^) effect on H_2_S donor-evoked vasoactive responses.

### 2.4. Total Activity of NO Synthase

**Figure 9:**
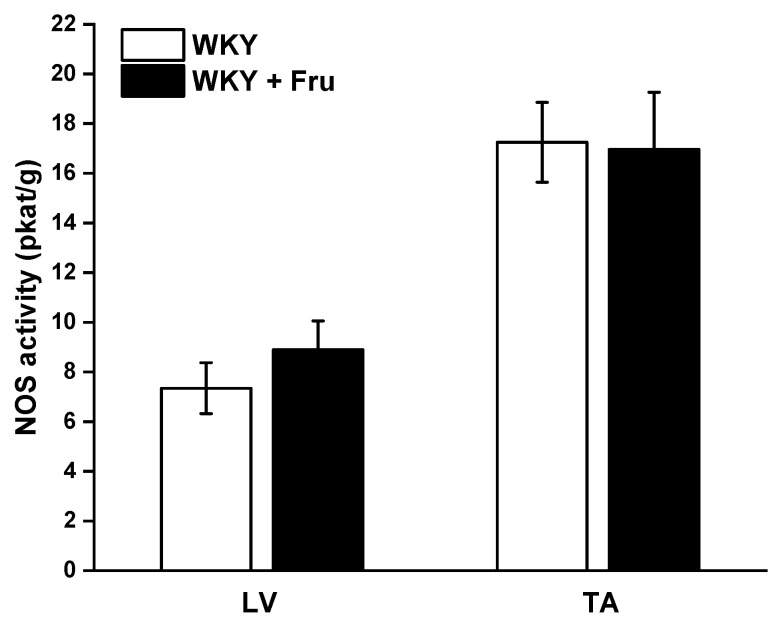
Total activity of NO synthase in different tissues. LV—left ventricle; TA—thoracic aorta; WKY—Wistar Kyoto rats; WKY + Fru—Wistar Kyoto rats treated with 10% fructose solution (*n* = 6). Data are expressed as the mean ± SEM.

The measurement of total NOS activity did not reveal any changes in either the left ventricle (LV) of the heart or the TA (Figure 9).

### 2.5. Expression of Enzymes That Produce NO and H_2_S

**Figure 10:**
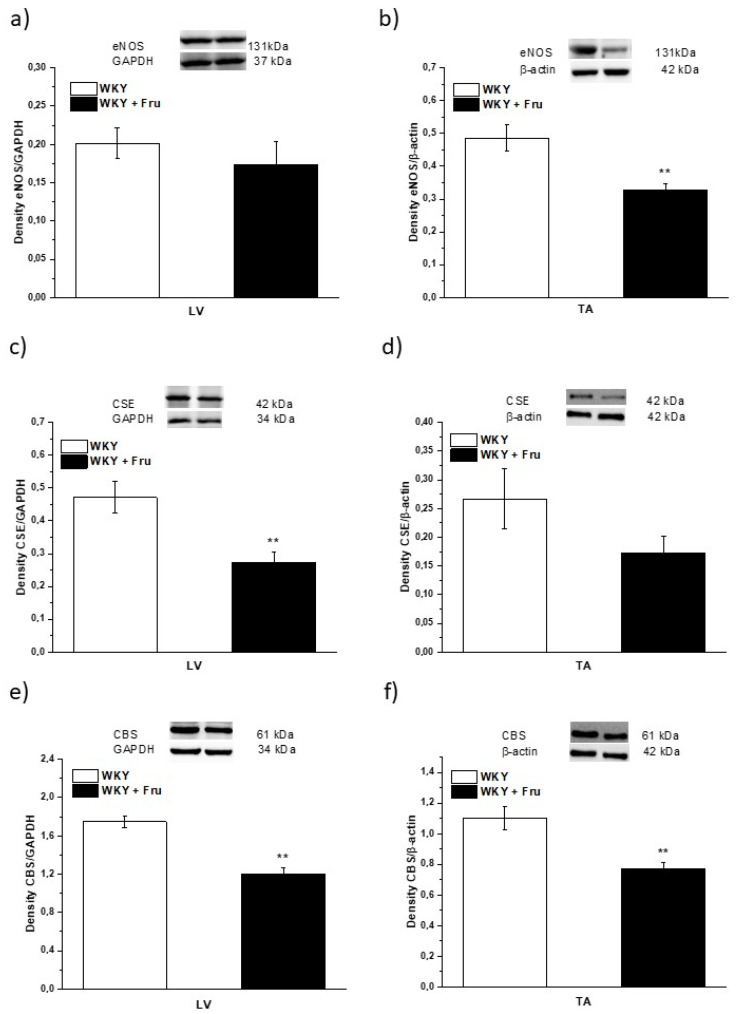
The expression of NO- and H_2_S-producing enzymes in different tissues. eNOS—endothelial NO-synthase (**a**,**b**); CSE—cystathionine γ-lyase (**c**,**d**); CBS—cystathionine β-synthase (**e**,**f**); LV—left ventricle (**a**,**c**,**e**); TA—thoracic aorta (**b**,**d**,**f**); WKY—Wistar Kyoto rats; WKY + Fru—Wistar Kyoto rats treated with 10% fructose solution (*n* = 6). Data are expressed as the mean ± SEM. ** *p* < 0.01 vs. WKY.

Our results demonstrated a significantly decreased expression of endothelial NO synthase (eNOS) in TA but not in LV (Figure 10a,b). The expression of cystathionine γ-lyase (CSE), which is considered a dominant producer of H_2_S in the arterial wall, was significantly reduced in the LV and there was a non-significant trend of decreased expression in the TA (*p* < 0.07; Figure 10c,d). The expression of another enzyme synthetizing H_2_S, cystathionine β-synthase (CBS), was confirmed by our measurement; moreover, it seems that CBS expression was probably higher than that of CSE. We observed significantly reduced CBS expression in both the TA and LV (Figure 10e,f).

## 3. Discussion

Metabolic syndrome is characterized by the coexistence of different severe abnormalities, including hypertension, abdominal obesity, hyperlipidemia, and disorders in insulin signaling. Several studies have confirmed that high fructose intake could alter pathologic processes in animal models as well as in humans, which triggered alterations coupled with metabolic syndrome [13,14]. However, according to other available studies, the question of a direct association of high fructose consumption with an increase in blood pressure represents a controversial issue. Chronic fructose administration significantly elevated SBP in normotensive conditions [1,7,15]; on the other hand, some authors found no impact of this treatment on blood pressure [16,17]. In our study, although we revealed a significant effect of the eight-week fructose administration on the values of systolic blood pressure, there were no differences between the experimental groups in individual weeks (Figure 1). In agreement with these results, we did not demonstrate any changes in heart trophicity. Moreover, we did not confirm obesity in rats treated with fructose (Table 1). This result could be influenced by a change in the food and liquid intake in the experimental groups. Although the rats receiving fructose had a higher liquid intake, their food intake was significantly lower than that of the control rats (Figure 2a,b). Similar results were demonstrated by Miranda et al. (2019) [3], who recorded increased liquid intake and energy intake but decreased food consumption throughout a 12-week-long period of 7% fructose solution treatment without any significant changes in the final body weight of normotensive Wistar rats. It has also been shown that fructose has a unique metabolism, occurring predominantly in the liver, and its long-term consumption is associated with hypertriglyceridemia [2]. In our experiments, the plasma level of triacylglycerols was not changed in fructose-treated animals; the levels of alanine aminotransferase and γ-glutamyl transferase were significantly increased, and there was a trend of increased levels of creatinine in this group, indicating damage to hepatocytes, liver disease, and kidney injury. According to our results, it seems that the eight-week-long fructose administration did not trigger disorders coupled with metabolic syndrome. We assume that these discrepancies are related to the different amounts of fructose consumed, the lengths of the treatment, and the ages and strains of the experimental animals. The experimental animals in this study were treated with 10% fructose solution, which represents a relatively low concentration of this saccharide; however, Miranda et al. (2019) [3] reported that the findings of studies using the highest fructose concentrations may not be compatible with human consumption. White et al. (2013) [18] also demonstrated that experimental rats tolerate high levels of fructose (≥60%), but that gastric stress can appear in humans even at low fructose doses (approximately 10%).

In our study, we confirmed that the long-lasting increase in fructose treatment did not affect the maximal force of adrenergic contraction but increased the sensitivity of adrenergic receptors to exogenous noradrenaline and significantly reduced endothelium-dependent vasorelaxation, although did not alter relaxation to NO donors (Figure 3a–d). Similar findings were observed by Zemancikova et al. (2014) [7], who reported an unchanged adrenergic contraction but decreased acetylcholine-induced vasorelaxation in the thoracic aorta of normotensive Wistar rats drinking 10% fructose solution. Moreover, they pointed out that fructose did not induce cardiovascular alterations in rats without a functional NO system in the state of NO deficiency (induced by N^G^-nitro-L-arginine methylester (LN) administration). According to these findings, they concluded that impairment of the NO system may be responsible for the adverse effects induced by high fructose intake. However, other studies did not report attenuated acetylcholine-induced relaxation after fructose treatment. Two-week-long administration of 20% fructose solution to Sprague Dawley rats had no impact on the contraction response to noradrenaline or the relaxant responses mediated by NO in the thoracic aorta [15]. Similarly, the response of the mesenteric vasculature to acetylcholine was unchanged in fructose-fed Wistar rats [2]. Moreover, Lozano et al. (2016) [19] demonstrated that NO-mediated relaxation was not affected by either a high-fructose or high-fat diet, or even simultaneous treatment, in the mesenteric arteries of normotensive rats. Despite the reduced NO-dependent vasorelaxation, our results demonstrate unchanged total NOS activity measured in the left heart ventricle (LV) and thoracic aorta (TA) of fructose-receiving rats. On the other hand, the protein expression of the eNOS isoform was reduced in the TA but comparable between the experimental groups in the LV (Figure 9 and Figure 10). Apart from eNOS, there are two other important isoforms of NOS in the cardiovascular system: the neuronal (nNOS) and inducible (iNOS) forms. The expression of all three NOS isoforms (eNOS, nNOS, and iNOS) was demonstrated not only in the intima, but also in the media of the TA and mesenteric and pulmonary arteries [20]. Moreover, it has been reported that nNOS and iNOS isoforms are able to produce vasoactively relevant NO to compensate for the downregulated eNOS/NO pathway [21,22,23]. Thus, the reduced eNOS expression in the TA could be compensated for by some of the other NOS isoforms to maintain the total activity of NOS. To evaluate the participation of the endogenous NO/NOS system in the regulation of vascular tone, we incubated isolated aortic rings with the non-specific NOS inhibitor LN. Acute LN treatment increased the contractile response to exogenous NA significantly more in fructose-fed rats compared to untreated rats (Figure 4b,c). On the other hand, we observed significantly inhibited endothelium-dependent vasorelaxation as a uniform vascular response to LN incubation. Both experimental groups had the same degree of inhibition, indicating a similar participation of acetylcholine-induced endogenous NO in the relaxation of the TA (Figure 4a). Based on these findings, it seems that fructose intake had a different impact on the participation of basic (independent of the receptor) and receptor-dependent NO production. While the receptor-independent NO component is able to significantly attenuate adrenergic contraction to the normal level, the inhibition of vasorelaxation mediated by receptor-dependent NO after fructose administration is probably not related to the deterioration of endothelial function, because at the level of NOS activity and the functional vasomotor responses, our results did not show any differences.

Our previous studies showed an obvious interaction between two important vasoregulatory molecules, NO and H_2_S, and their pathways and suggested the existence of a coupled nitroso–sulfide signaling pathway [24,25]. Herein, we confirmed that the heterogeneity of specific nitroso–sulfide vasoactive signaling exists depending on the occurrence of different pathological stages, e.g., hypertension or increased plasma glucose levels [26]. Recently, in a non-obese rat model of metabolic syndrome (HTG rats), we confirmed a significantly higher expression of CSE in the arterial wall of the TA compared to control rats, and that the endogenous H_2_S participated in the inhibition of NO-mediated vasorelaxation [27]. Thus, based on these results, it seems that the presence of inherent metabolic disturbances could significantly affect nitroso and sulfide signaling and their mutual interaction. The aortic tissue has been shown to have much higher H_2_S levels than other vascular tissues [28]; therefore, in the next part of our study, we focused on the question of whether fructose intake was able to modulate the participation of the nitroso–sulfide signaling pathway in the vasoregulation of the TA. We found that the incubation of aortic rings with L-cysteine, a precursor of endogenous H_2_S production, and pyridoxal 5′-phosphate, a cofactor of CSE and CBS, increased the maximal force of NA-induced contraction in the control group but had no impact on the vasorelaxation of the TA (Figure 5). However, a H_2_S scavenger, bismuth (III) subsalicylate (BSC), restored the increased sensitivity of adrenergic receptors in fructose-fed rats to a level similar to that in control rats. In addition, BSC (similarly to propargylglycine, a CSE inhibitor) reduced endothelium-dependent vasorelaxation in control rats (Figure 6 and Figure 7). The elimination of H_2_S by using a scavenger in the control group reduced endothelium-dependent vasorelaxation in the same manner as chronic fructose intake. Therefore, the results could indicate that the observed deterioration of endothelial function in fructose-fed rats was probably associated with the absence of a synergistic involvement of endogenous H_2_S in NO-mediated vasorelaxation. Moreover, H_2_S itself could probably participate in endothelial dysfunction in these rats because BSC significantly increased the vasorelaxant response in rats receiving fructose solution. The synergistic effect of NO and H_2_S has been suggested by several studies. Coletta et al. (2012) [29] also showed that NO and H_2_S are mutually required for the physiological control of vascular function. The authors confirmed that pre-treatment with a low concentration of NaHS (30 μmol/L, 15 min) potentiated the vasorelaxant response of the TA to acetylcholine and to the NO donor, which was accompanied by significantly increased cGMP levels. Chen et al. (2014) [30] demonstrated that the application of a H_2_S donor increases the expression of eNOS and upregulates eNOS activity due to the phosphorylation of its serine residue. Moreover, the determination of the expression of CSE and CBS in our study also confirmed a significant effect of high fructose intake on endogenous H_2_S synthesis. In the LV, the expression of both CSE and CBS was reduced after fructose treatment, while in the TA, the expression of CBS was found to be significantly lower and there was a non-significant trend of the decreased CSE expression in the TA. Interestingly, it seems that there was a higher expression of CBS than in CSE in the aortic arterial wall (Figure 10). We previously found similar results in human intrarenal arteries, where we did not record gene expression of CSE in the arterial wall, but the mRNA of CBS was detectable [26]. This finding could explain the evidence that the acute inhibition of CSE revealed weaker vasoactive effects than the H_2_S scavenger, and BSC could eliminate H_2_S also produced by CSE-independent sources, including CBS. According to our results, it seems that not only CSE, which is considered a dominant H_2_S producer in the cardiovascular system, but also CBS significantly contributes to the vasoactive manifestation of endogenous H_2_S.

Recent studies suggested H_2_S, through application of its donors or as an added substance, as a novel potential pharmacological tool in different pathological circumstances [31,32,33,34]. In our previous papers, we showed that in both renal arteries of spontaneously hypertensive rats and internal lobar arteries of hypertensive patients, the simultaneous application of NO and H_2_S donors evoked a specific vasoactive effect (enhanced maximum relaxation and changed dynamics, respectively), thus demonstrating the importance of H_2_S–NO interactions [35]. Application of a H_2_S donor predominantly induces a biphasic vasoactive response [36,37], which was also confirmed by our results (Figure 8a,b). We did not record a significant effect of increased fructose intake on this response, and in both groups, Na_2_S-induced relaxation was primarily associated with hyperpolarization via the activation of K_ATP_ channels, as suggested by Zhao et al. (2011) [38]. According to our previous results, we suggested that during acute NO deficiency, H_2_S donors are able to exert enhanced vasorelaxation to maintain the vasorelaxant capacity of the arterial wall. Moreover, the response was strengthened in a state of arterial hypertension, which we considered a reserve mechanism of the TA to compensate for the pathological processes [36,37]. We also showed that the higher level of glucose in the plasma of patients modified the vasoactive response of the products of the nitroso–sulfide signaling pathway [26]. Similarly, in this study, fructose treatment changed the participation of the NO system in the vasoactive effect of the H_2_S donor. Fructose intake diminished the increased vasorelaxant effect of H_2_S donors observed during acute NO deficiency. Based on our results, it seems that a modification of saccharide metabolism (increased glycaemia or increased fructose intake) could significantly alter the interaction between the H_2_S donor and (endogenous) NO. To the best of our knowledge, findings emphasizing the crucial role of the mutual interaction between the nitroso and sulfide signaling pathways in fructose-related pathologies have not previously been reported.

## 4. Materials and Methods

### 4.1. Guide for the Use and Care of Laboratory Animals

Procedures were performed in accordance with the guidelines of the Institute of Normal and Pathological Physiology, Centre Experimental Medicine Slovak Academy of Sciences (INPP CEM SAS), and were approved by the State Veterinary and Food Administration of the Slovak Republic and by an ethics committee according to the European Convention for the Protection of Vertebrate Animals used for Experimental and Other Scientific Purposes, Directive 2010/63/EU of the European Parliament(08/01/2019). All rats were housed under a 12 h light–12 h dark cycle at a constant humidity (45–65%) and temperature (20–22 °C). Animals were provided INPP CEM SAS veterinary care. Twelve-week-old male Wistar Kyoto (WKY) rats were used in this study (*n* = 12). The animals were randomly divided into two groups of six animals each: control WKY receiving tap water, and WKY receiving eight weeks of 10% fructose solution in their drinking water. Both groups had free access to identical standard laboratory rat chow.

### 4.2. Basic Cardiovascular and Biometric Parameters

The liquid and food intakes were monitored daily. During the 8 weeks of the experiment, systolic blood pressure (SBP) was measured by a non-invasive plethysmography method once a week. To follow the effect of fructose intake on body weight (BW), we recorded it weekly, and then the final BW was determined before decapitation after brief anaesthetization with CO_2_. The weight of the heart (HW) and retroperitoneal adipose tissue, and the length of the tibia, were recorded for the calculation of biometric parameters such as cardiac and renal hypertrophy and adiposity.

### 4.3. Collection of Plasma Samples

Plasma samples were collected just after decapitation and frozen in aliquots for biochemical determinations. The basic levels of triacylglycerols (TAG), high-density lipoprotein cholesterol (HDL), alanine aminotransferase (ALT), gamma glutamyl transferase (γ-GT), cystatin C (CYS), creatinine (CRE), and glucose (GLU) were commercially determined at the Laboklin GMBH. The values of parameters represented pre-prandial concentrations.

### 4.4. Functional Study

Although the TA is a large conduit artery, to avoid impairment of the endothelium, the TA was carefully cleaned of connective tissue and cut into 5 mm-long rings using a binocular microscope. The rings were vertically fixed between 2 stainless wire triangles and immersed in a 20 ml incubation organ bath with oxygenated (95% O_2_; 5% CO_2_) Krebs solution (118 mmol/L NaCl; 5 mmol/L KCl; 25 mmol/L NaHCO_3_; 1.2 mmol/L MgSO_4_.7H_2_O; 1.2 mmol/L KH_2_PO_4_; 2.5 mmol/L CaCl_2_; 11 mmol/L glucose; 0.032 mmol/L CaNa_2_EDTA) kept at 37 °C. The upper triangles were connected to sensors of isometric tension (FSG-01), and the changes in this tension were registered by AD converter Advanced Kymograph software (all from MDE, Budapest, Hungary). A resting tension of 1 g was applied to each ring and maintained throughout a 45–60 min equilibration period.

#### Experimental Protocol

Briefly, concentration-dependent contractile responses were induced by increasing doses of noradrenaline (NA; 10^−10^ to3 × 10^−5^ mol/L) in a cumulative manner. Contractions were expressed as developed tension (g) or as a percentage of the maximum tissue responses to the agonist (demonstrating the sensitivity of adrenergic receptors). The relaxant responses were followed on rings pre-contracted with noradrenaline, and after the achievement of a stable plateau, the preparations were exposed to cumulative doses of acetylcholine (Ach; 10^−10^ to 3 × 10^−5^ mol/L) and sodium nitroprusside (10^−10^ to 3 × 10^−5^ mol/L). The rate of relaxation was expressed as a percentage of the noradrenaline-induced contraction. We evaluated the effect of the non-specific inhibitor of NOS, N^G^-nitro-L-arginine methylester (LN; 10^−4^ mol/L); the specific CSE inhibitor, DL-propargylglycine (PPG; 10^−2^ mol/L); the H_2_S scavenger, bismuth(III) subsalicylate (BSC; 10^−6^ mol/L); and pyridoxal 5′-phosphate hydrate (PLP; 2 × 10^−3^ mol/L) and L-cysteine (L-Cys; 5 × 10^−3^ mol/L) as precursors of H_2_S production on NA-induced contraction and Ach-induced relaxation. All drugs were acutely incubated for 20 min in the organ bath.

Na_2_S•9H_2_O was used as a H_2_S donor that dissociates in water solution to Na^+^ and S^2−^, which reacts with H^+^ to yield HS^−^ and H_2_S. We use the term Na_2_S to encompass the total mixture of H_2_S, HS^−^ and S^2−^. Direct vasoactive effects of Na_2_S were observed on NA-pre-contracted (10^−6^ mol/L) rings by the administration of increasing doses of Na_2_S (20, 40, 80, 100, 200, 400 μmol/L). The participation of the endogenous NO system in the vasomotor responses of TA was followed before and 20 minutes after pre-treatment with a non-specific inhibitor of NOS, NG-nitro-L-arginine methyl ester (L-NAME; 10^−4^ mol/L), to block basal and receptor-induced endogenous NO production. Moreover, glibenclamide (GLI; 10^−4^ mol/L) was used to demonstrate the participation of K_ATP_ channels in these vasoactive responses.

### 4.5. Measurement of the Total NO Synthase Activity

Total NOS activity was determined in crude homogenates of the aorta and left ventricle by measuring the formation of [3H]-L-citrulline from [3H]-L-arginine (ARC, St. Louis, MT, USA), as previously described and slightly modified by Pechanova et al. [39]. [3H]-L-Citrulline was measured with a Quanta Smart TriCarb Liquid Scintillation Analyzer (Perkin-Elmer, Meriden, CT, USA). NOS activity was expressed as pkat/min per gram of protein.

### 4.6. Expression of Enzymes Producing NO and H_2_S

Protein expression of eNOS, CBS and CSE was determined in the aorta and left ventricle by Western blot analysis. The samples were probed with primary rabbit polyclonal anti-eNOS (Abcam, Cambridge, UK) and anti-CBS (Proteintech, Manchester, UK) and mouse monoclonal anti-CSE (Proteintech, Manchester, UK) antibodies. Anti-GAPDH and anti-β-actin (Abcam, Cambridge, UK) were used as loading controls for the left ventricle and aorta, respectively. A chemiluminescence ECL system (Bio-Rad, Hercules, CA, USA) was used for band intensity visualization. The ChemiDocTM Touch Imagine System (Image Lab^TM^ Touch software, version 5.2, Bio-Rad, Hercules, CA, USA) was used for quantification of the bands.

### 4.7. Statistical Analyses

The data are expressed as the mean ± S.E.M. For the statistical evaluation of vasoactive responses between groups, three-way analysis of variance (ANOVA) with the Bonferroni post hoc test was used. To evaluate general cardiovascular and plasma parameters, including NO synthase activity and eNOS, CSE, and CBS expression, one-way ANOVA was used with the Bonferroni post hoc test. Differences between means were considered significant at *p* < 0.05.

## 5. Conclusions

In summary, the present results demonstrated a special interaction between the nitroso and sulfide signaling pathways in fructose-fed normotensive rats. Although chronic increased fructose intake did not initiate changes characterizing metabolic syndrome, it impaired endothelium-dependent vasorelaxation, predicting possible cardiovascular complications. Moreover, based on our results, we could assume that this reduction in vasorelaxation in the thoracic aorta was probably not directly associated with the decreased production of NO, but rather with impairment of the NO–H_2_S interaction. We confirmed that fructose altered the vasomotor manifestation of this interaction at least at two different levels: (i) the contribution of endogenous H_2_S to NO-mediated vasorelaxation; and (ii) the contribution of endogenous NO to the vasoactive effect of the H_2_S donor.

## Figures and Tables

**Table 1:** General parameters of the experimental animals.

	*n*	WG (g)	HW/BW (mg/g)	HW/TL (mg/mm)	RW/TL (mg/mm)
WKY	6	78.7 ± 7.91	3.37 ± 0.1	29.7 ± 2.2	96.5 ± 16.9
WKY + FRU	6	80.5 ± 8.7	3.49 ± 0.1	31.4 ± 1.1	82.8 ± 14.8

WKY—Wistar Kyoto rats; WKY + FRU—Wistar Kyoto rats treated with 10% fructose solution; WG—weight gain; HW/BW—ratio of the heart weight to body weight; HW/TL—ratio of the heart weight to tibia length; RW/TL—ratio of the retroperitoneal adipose tissue weight to tibia length. Data are expressed as the mean ± SEM.

**Table 2:** The basic plasma parameters.

	TAG mmol/L	HDL mg/dL	ALT U/L	y-GT U/L	CYS mg/L	CRE µmol/L	GLU mmol/L
WKY	1.05 ± 0.35	62.18 ± 6.07	18.72 ± 2.25	0.17 ± 0.08	1.98 ± 0.16	44.2 ± 4.04	9.28 ± 1.12
WKY + FRU	1.18 ± 0.21	68.83 ± 4.59	31.95 ± 4.75 *	0.62 ± 0.16 *	2.15 ± 0.02	54.17 ± 3.1	10.63 ± 0.54

WKY—Wistar Kyoto rats; WKY + FRU—Wistar Kyoto rats treated with 10% fructose solution; TAG—triacylglycerols; HDL–high density cholesterol; ALT—alanine aminotransferase; γ-GT-gamma glutamyl transferase; CYS—cystatin C; CRE—creatinine; GLU—glucose (*n* = 6). Values are mean ± S.E.M * *p* < 0.05 vs. WKY.

## Data Availability

All data arising from this study are contained within the article and any additional data sharing will be considered by the first author upon request.
